# Protective Effect of Hemoglobin Treatment with Sodium Trimetaphosphate on Enamel Erosion: *in vitro* study

**DOI:** 10.1590/0103-644020256623

**Published:** 2025-12-01

**Authors:** Carolina Ruis Ferrari, Karolyne Sayuri de Araujo Kitamoto, Monique Malta Francese, Vinícius Taioqui Pelá, João Victor Frazão Câmara, Lethycia Almeida Santos, Adrian Lussi, Tamires Passadori Martins, Juliano Pelim Pessan, Marília Afonso Rabelo Buzalaf

**Affiliations:** 1 Department of Biological Sciences, Bauru School of Dentistry, University of São Paulo, Bauru, São Paulo, Brazil.; 2 University Hospital for Conservative Dentistry and Periodontology, Medical University of Innsbruck, Innsbruck, Austria and Department of Restorative, Preventive and Pediatric Dentistry, School of Dental Medicine, Bern, Switzerland.; 3 Department of Preventive and Restorative Dentistry, School of Dentistry, São Paulo State University(Unesp), Araçatuba, São Paulo, Brazil.

**Keywords:** acquired enamel pellicle, hemoglobin, intrinsic dental erosion, pellicle modification, sodium trimetaphosphate

## Abstract

This study aimed to evaluate the effect of hemoglobin (Hb) in combination with different concentrations of sodium trimetaphosphate (STMP) on initial enamel erosion *in vitro*. Ninety bovine enamel samples (4X4mm) were prepared and divided into six groups (n/group=15), according to the treatments: 1) Deionized water (negative control); 2) Commercial solution Elmex Erosion Protection® ; 3) 1.0 mg/mL Hb; 4) 1.0 mg/mL Hb+0.5% STMP; 5) 1.0 mg/mL Hb+1.0% STMP; 6) 1.0 mg/mL Hb+3.0% STMP. The samples were treated with the respective solutions (250µL, 2h, 37ºC, constant agitation), and the acquired enamel pellicle was formed for 2h using pooled stimulated human saliva. Subsequently, the samples underwent an erosive challenge with 0.01 M HCl (pH 2.3, for 10 seconds) to simulate intrinsic erosion. These procedures were performed once/day for 3 consecutive days. Demineralization was assessed by percentage surface hardness change (%SHC) and the relative surface reflection intensity (%SRI) by the Reflectometer Optipen. Data were analyzed by ANOVA and Tukey's tests (p < 0.05). The negative control exhibited significantly less protection for both variables compared to the other groups. Regarding %SHC, the most effective treatments were 1.0 mg/mL Hb + 0.5% STMP and 1.0 mg/mL Hb + 1.0% STMP, which surpassed all other groups, including the positive control. The best protection for %SRI was observed with 1.0 mg/mL Hb. These results suggest that combining Hb with an inorganic component may support the development of strategies to prevent intrinsic enamel erosion.

## Resumo

Este estudo teve como objetivo avaliar o efeito da hemoglobina (Hb) em combinação com diferentes concentrações de trimetafosfato de sódio (STMP) na erosão inicial do esmalte *in vitro*. Noventa amostras de esmalte bovino (4x4mm) foram preparadas e divididas em 6 grupos (n/grupo=15), de acordo com os tratamentos: 1) Água deionizada (controle negativo); 2) Solução comercial Elmex Erosion Protection®; 3) 1,0 mg/mL de Hb; 4) 1,0 mg/mL de Hb+0,5% de STMP; 5) 1,0 mg/mL de Hb+1,0% de STMP; 6) 1,0 mg/mL de Hb+3,0% de STMP. As amostras foram tratadas com as respectivas soluções (250µL, 2h, 37ºC, agitação constante) e a película de esmalte adquirida foi formada ao longo de 2h utilizando saliva humana estimulada. Posteriormente, as amostras foram submetidas a desafio erosivo com HCl 0,01 M (pH 2,3, por 10s) para simular erosão intrínseca, uma vez ao dia, durante 3 dias consecutivos. A desmineralização foi avaliada pela porcentagem de variação da dureza superficial (%SHC) e pela intensidade relativa de reflexão superficial (%SRI) pelo Reflectômetro Optipen. Os dados foram analisados ​​pelos testes ANOVA e Tukey (p < 0,05). O controle negativo apresentou proteção significativamente menor para ambas as variáveis ​​em comparação aos demais grupos. Em relação à %SHC, o tratamento mais eficaz foi 1,0 mg/mL de Hb + 0,5% de STMP e Hb + 1,0% de STMP, superando todos os grupos, incluindo o controle positivo. A melhor proteção para %SRI foi observada com 1,0 mg/mL de Hb. Esses resultados sugerem que a combinação de Hb com um componente inorgânico pode contribuir para o desenvolvimento de estratégias de prevenção da erosão intrínseca do esmalte.

**Figure:**
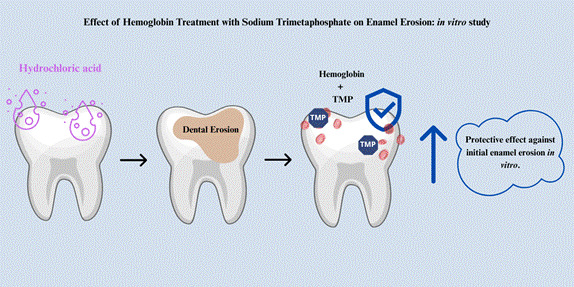

## Introduction

Erosive tooth wear is characterized by the cumulative loss of hard dental tissue, an irreversible process, caused by the combination of physical and/or chemical-physical processes, with erosion as the primary causative factor ^1^. Erosion is characterized by the chemical loss of mineralized substance due to exposure to non-bacterial acids, originating from the diet (extrinsic) or from the host's gastric contents (intrinsic) ^1^.

Intrinsic dental erosion warrants attention due to the factors contributing to this condition, which involve the presence of gastric acids in the oral cavity that exceeds the buffering capacity of saliva ^2^. This occurs in situations such as the presence of gastroesophageal reflux disease, nervous bulimia, chronic alcoholism, and hyperemesis gravidarum in pregnant women ^2^. Additionally, gastroesophageal reflux disease affects 13-19% of people worldwide, making it the second most common gastrointestinal disorder ^3^, which impacts the quality of life of patients who may complain of dental sensitivity, yellowish discoloration of the teeth, and a lack of aesthetics ^4^.

Saliva plays a crucial role in erosion due to its diverse functions, including protection of the teeth, achieved through the formation of the acquired enamel pellicle ^5^. This pellicle is characterized as an organic layer, free of bacteria, that forms *in vivo* due to the selective adsorption of salivary proteins to the dental surface ^5^ ^,^ ^6^. Among the various proteins that compose the acquired enamel pellicle, hemoglobin (Hb) has been identified and demonstrated to exhibit resistance against erosive tooth wear ^7^. In addition, several Hb subunits were found to have an expression more than three times higher in patients with gastroesophageal reflux disease and without erosion, compared to those with the same disease but presenting erosive lesions ^8^. The Hb exhibits a strong affinity for hydroxyapatite, and its adsorption rate to hydroxyapatite increases as the pH decreases, probably because the isoelectric point of this protein is around 6.8-7.0, meaning it becomes positively charged when the pH is less than 6.8 ^9^ ^,^ ^10^.

New preventive components against erosion, such as the addition of sodium trimetaphosphate (STMP) to fluoridated vehicles, have been studied and shown to significantly enhance their ability to mitigate enamel demineralization caused by cariogenic and erosive challenges, even when combined with varnishes containing low fluoride content ^11^ ^,^ ^12^. These effects are attributed to STMP's high adsorption capacity to enamel, which consequently reduces its solubility and exhibits a strong tendency to form complexes with cations due to the presence of a phosphate group in STMP's molecular structure ^13^ ^,^ ^14^.

Despite the lack of data on STMP’s effects on acquired enamel pellicle, evidence indicates that the effects of this phosphate on enamel are related to influencing the bond strength with proteins, due to its high adsorption capacity to enamel ^13^ ^,^ ^14^ and to its ability to alter the surface free energy of this substrate ^15^. Based on these findings, it is plausible to assume that a similar effect may occur for the association between Hb and STMP concerning the protection of enamel against initial intrinsic erosive challenges in dental enamel, as Hb, when present in acquired enamel pellicle, resisted removal by hydrochloric acid (HCl) ^8^. Current preventive measures for intrinsic enamel erosion have limitations, including low efficacy against intrinsic acids and a limited emphasis on modulating the acquired enamel pellicle. Therefore, there is a clinical need for innovative strategies that enhance the protective effect on enamel, particularly by combining organic and inorganic components.

Therefore, the objective of this study was to evaluate the protective effect of Hb associated with STMP against initial intrinsic enamel erosion *in vitro*. The experimental hypothesis was that the combination of Hb and STMP would enhance the protection of enamel against initial intrinsic erosive challenges compared to Hb used alone. The null hypothesis tested was that Hb associated with STMP did not protect against enamel erosion *in vitro.*

## Materials and methods

### Ethical aspects

This research was approved by the Bauru School of Dentistry Animal Research Ethics Committee under number 017/2023 and the Bauru School of Dentistry Human Research Ethics Committee under number CAAE: 75832423.1.0000.5417.

### Sample calculation and preparation

The sample calculation was based on the study by Santiago et al. ^16^. A minimum detectable difference in the percentage of surface hardness change (%SHC) of 27% was considered, taking into account a standard deviation of 9%, an α error of 5% and a β error of 20%. For this purpose, 15 specimens were calculated.

Ninety enamel specimens (4x4 mm) were prepared from the buccal surface of bovine incisors. The crowns of the bovine teeth were individually attached to acrylic plates (40x40x5 mm^3^) and secured into an ISOMET Low Speed Saw precision cutting device (Buehler, Lake Bluff, USA). With two double-sided diamond discs - XL 12205, “High concentration”, 102 x 0.3 x 12.7 mm^3^ (Extec Corp., Enfield, CT, USA/Ref: 12205) and a stainless-steel spacer (diameter 7 cm, thickness 4 mm and central hole 1.3 cm) between the discs, the specimens were cut. Following this, they were polished with silicon carbide sandpaper, grade 320-600-1200 (Buehler), cooled with deionized water. The specimens were cleaned in distilled water using an ultrasonic bath and then stored at 4°C until the start of the experiments. 

### Human saliva collection

Nine volunteers, aged 20-35 years (four men and five women), were selected for saliva collection. All research participants signed the informed consent form, as agreed by the ethics committee of the Bauru School of Dentistry. The exclusion criteria for general health were pregnant women, smokers, use of prolonged medication, and those with systemic diseases. Regarding oral health, the exclusion criteria were active caries, periodontal disease, and erosive tooth wear. The stimulated and unstimulated salivary flows were greater than 1 and 0.3 mL/min, respectively. The stimulated saliva was collected during the morning (9-10 a.m.), using Paraffin wax for 10 min, under temperature control (the tube for saliva collection was kept on ice). After all, the saliva was pooled, and the supernatants were separated by centrifugation (14,000 g, 20 min, at 4 °C). Finally, the saliva was aliquoted (for each day of the experiment) and stored at -80 °C.

### Treatment Groups

The Hb (1.0 mg/mL) used in this study was human (Hb, Sigma Aldrich, #H7379), as reported in a previous study ^7^. Enamel specimens were randomly divided into 6 groups (n=15/group), as follows:

1. Deionized water (negative control);
2. Commercial solution Elmex Erosion Protection ® (Gaba International AG; positive control);
3. 1.0 mg/mL Hb;
4. 1.0 mg/mL Hb + 0.5% STMP;
5. 1.0 mg/mL Hb + 1.0% STMP;
6. 1.0 mg/mL Hb + 3.0% STMP.

### Experimental procedures

**Figure 1:**
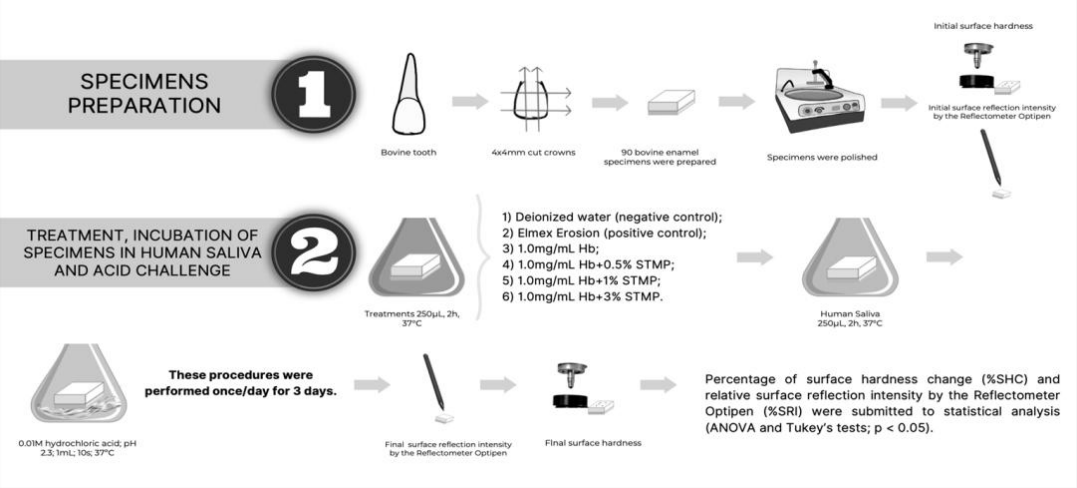
Experimental Procedures Protocol: Ninety bovine incisor enamel samples (4x4 mm) were prepared (n = 15/group). Initially, the hardness analysis and surface reflection intensity were assessed using a hand-held Reflectometer Optipen. The samples were then individually treated according to their respective groups for 2 hours. Subsequently, the samples were incubated in saliva for 2 hours. The erosive challenge was conducted using HCl for 10 seconds. These procedures were repeated daily for 3 consecutive days, and the final analysis was then performed.

The protocol used was modified based on the study by Cheaib and Lussi (2011) ^17^ and is illustrated in Figure 1. The samples were treated (individually) according to the groups described above (250 μL, 2 h, 37° C, constant agitation). Subsequently, the acquired enamel pellicle was formed by adding pooled human saliva to each sample (250 μL, 2 h, 37 °C, constant shaking). Following the formation of acquired enamel pellicle, the samples were individually subjected to an erosive challenge in 0.01 M HCl (1 mL, pH 2.3, for 10 s, 37 °C), simulating intrinsic erosion. The samples were washed (10 s) carefully between each application (treatment, pellicle formation, and acid challenge). These procedures were performed once a day, for 3 consecutive days.

### Hardness analysis

The surface hardness (SH) of the enamel specimens was measured using a Hardness Tester (SH-HMV-2000; Shimadzu, Kyoto, Japan) with a Knoop diamond. Five indentations per sample were made (at intervals of 150 µm between them and in the center of the sample) at the beginning of the experiment (SH initial), and after the experimental procedures (SH final). A load of 50 g and a dwell time of 15 s was used. The data were organized as a percentage of surface hardness change (% SHC), according to the following equation: % SHC = ([SH initial - SH final] / SH initial) × 100.

### Measurement by the reflectometer Optipen

The Surface Reflection Intensity (SRI) was measured using a handheld Reflectometer Optipen. The baseline (SRIb) was made before treatment, and the final analysis (SRIf) was made after 3 consecutive days of the erosive challenge. For both analyses (baseline and final), all enamel surfaces were previously dried for 3 seconds, and the tip of the reflectometer was gently touched to the enamel surface. Then, the portable equipment was inclined at various angles to obtain the highest reflection record of each tooth surface area. The values presented by the software were tabulated and calculated as follows: %SRI = (SRI_f_ / SRI_b_) × 100 ^18^.

### Statistical analysis

GraphPad Prism software (version 6.0 for Windows; GraphPad Software Inc., La Jolla, CA, USA) was used. Data were checked for normality (Shapiro-Wilk test) and were analyzed by ANOVA, followed by Tukey's multiple comparison test. Results are presented as mean±standard deviation. The significance level was set at 5%. 

## Results

### Percentage of Surface Hardness Change on Enamel

**Figure 2:**
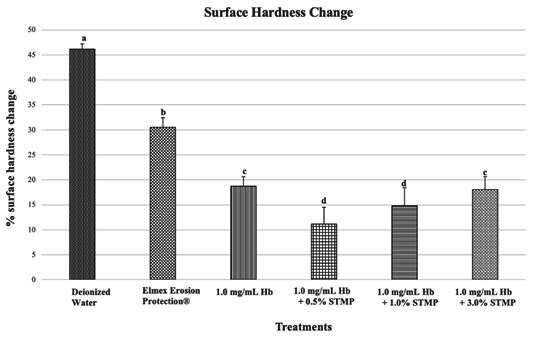
Percentage of surface hardness change after 3 consecutive days of treatments of bovine enamel specimens with Deionized water (negative control), Elmex Erosion Protection® (positive control), 1.0 mg/mL Hb, 1.0 mg/mL Hb + 0.5% STMP, 1.0 mg/mL Hb + 1.0% STMP, 1.0 mg/mL Hb + 3.0% STMP, followed by the formation of the acquired enamel pellicle for 2 h and erosive challenge with 0.01 M HCl (pH 2.3) for 10 s. Distinct letters show significant differences among the treatments (ANOVA and Tukey's multiple comparison test, p < 0.05, n = 15/group).

Figure 2 shows the percentage surface hardness change (% SHC) according to the different groups. In general, Hb and all combinations with STMP significantly reduced the initial enamel erosion when compared to the negative control and positive control (p < 0.05). The most effective treatments were 1.0 mg/mL Hb + 0.5% STMP and 1.0 mg/mL Hb + 1.0% STMP. The protection conferred by the negative control (46.16±1.0 %) was significantly lower in comparison with the other groups [Elmex Erosion Protection® (30.50±1.94 %), 1.0 mg/mL Hb (18.71±1.94 %), 1.0 mg/mL Hb + 0.5% STMP (12.43±1.70 %), 1.0 mg/mL Hb + 1.0% STMP (14.78±3.59 %) and 1.0 mg/mL Hb + 3.0% STMP (18.00±2.59 %)].

### Enamel loss measured by Reflectometer Optipen

**Figure 3:**
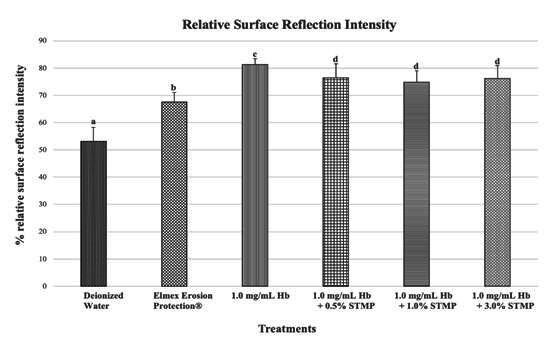
Relative Surface reflection intensity (%SRI) according to the different groups: Deionized water (negative control), Elmex Erosion Protection® (positive control), 1.0 mg/mL Hb, 1.0 mg/mL Hb + 0.5% STMP, 1.0 mg/mL Hb + 1.0% STMP, 1.0 mg/mL Hb + 3.0% STMP. Distinct letters mean significant differences between treatments (ANOVA and Tukey's multiple comparison test, p < 0.05, n = 15/group).

The %SRI results (mean±standard deviation) showed that the protection conferred by the deionized water group (negative control) (53.08±5.16 %) was significantly lower in comparison with the other groups [Elmex Erosion Protection® (67.52±3.59 %), 1.0 mg/mL Hb (81.32±2.13 %), 1.0 mg/mL Hb + 0.5% STMP (76.49±5.12 %), 1.0 mg/mL Hb + 1.0% STMP (74.78±4.28 %) and 1.0 mg/mL Hb + 3.0% STMP (76.23±4.74 %)]. The best protection (least change in reflectivity and presenting higher %SRI values) was observed for 1.0 mg/ mL Hb, performing better than the gold standard (Commercial solution with SnCl_2_/NaF/AmF) (p < 0.05) (Figure 3).

## Discussion

Despite inorganic components being the most studied strategies for preventing dental erosion, previous *in vitro* and *in situ* studies have shown that STMP is effective in de- and remineralizing enamel ^19^ ^,^ ^20^. Within this context, considering the effectiveness of the organic component (Hb) against dental erosion ^7^ and the favorable role of STMP ^(^ ^20^ ^,^ ^21^ ^,^ ^22^ ^,^ ^23^, it is important to test the combination of both components in a solution to verify their synergistic preventive effect against dental erosion.

To the best of our knowledge, this is the first study capable of analyzing the effectiveness of the Reflectometer Optipen *in vitro* using the Hb solution as a preventive agent for initial dental erosion. For this, the present study was designed as a proof-of-concept for previous *in vivo* results involving CaneCPI-5 ^24^, with the aim of developing a clinical protocol for the use of a rinse solution containing Hb in future studies.

We have identified significant alterations in the protein profile of the acquired enamel pellicle in patients with gastroesophageal reflux disease and erosive tooth wear, compared to those without this buccal condition ^(^ ^8^. Interestingly, the proteome of the acquired enamel pellicle of gastroesophageal reflux disease patients without erosive tooth wear showed an increased expression of various subunits of Hb, different isoforms of cystatin, as well as albumin, when compared to gastroesophageal reflux disease patients with erosive tooth wear ^(^ ^8^. Among these proteins, the most significant increase was found for Hb (more than 3-fold).

Hemoglobin is a large and complex protein molecule with the primary function of transporting oxygen from the lungs to the tissues. Its affinity for hydroxyapatite has long been recognized, with hydroxyapatite columns demonstrating excellent performance in purifying Hb ^7^. Considering this, hydroxyapatite microspheres or polyhedral have been developed for the controlled delivery of this protein ^10^. Interestingly, the adsorption of Hb to hydroxyapatite increases as the pH decreases, a phenomenon attributed to electrostatic interactions between Hb molecules and hydroxyapatite, mediated by van der Waals forces, electrostatic, or hydrophobic interactions ^10^. Our research group has employed novel approaches to enhance the adsorption of human Hb, focusing on acquired pellicle engineering. This procedure involves the application of the protein solution before the formation of the acquired enamel pellicle, potentially enhancing the level of prevention against erosion. Furthermore, studies investigating this concept in the field of proteomic analysis have shown that *in vivo* treatment with Hb rinse increases the concentration of important pellicle proteins ^25^.

Several methodological aspects merit consideration. The Hb at a concentration of 1.0 mg/mL was used in the present study based on findings by Martini et al. ^7^, which demonstrated a reduction in the percentage of surface hardness compared to the negative control. This was assessed using an *in vitro* model of initial erosion developed by Cheaib and Lussi ^17^, adapted to evaluate intrinsic erosion. The main novelties of the present study were: 1) the utilization of the Reflectometer Optipen in an *in vitro* protocol of initial erosion with treatment combining Hb and STMP, and 2) reinforcing the acquired enamel pellicle with Hb associated with an inorganic component. The reflectometer is a compact, pen-size device that provides easy handling and does not require significant laboratory space. The illumination is provided by a halogen lamp, and the measurement is made using a diode laser beam (wavelength: 635 nm) with an angle of incidence and reflection of 23 degrees. The reflected light is then captured and measured with a photodiode (FDS100, Thorlabs, Dachau, Germany).

The results obtained in the present study with the Hb solution represent a significant advancement in the prevention of initial erosion, as the protein treatment outperformed the gold standard (positive control group, commercial solution) in this study. It is worth noting that the reflection on enamel may have been influenced by two factors: 1) the strong binding of Hb to hydroxyapatite, which could have contributed to increased reflection and, consequently, enhanced enamel protection; and 2) the commercial solution, which has the potential to interact with the acquired enamel pellicle and alter it, resulting in a higher abundance of proteins and increased reflection. However, several potential limitations regarding the use of Hb as a preventive agent may be considered. These include concerns about the stability of this in solution, particularly during prolonged storage or within the oral environment, which may influence its effectiveness. Moreover, due to its characteristic reddish coloration, hemoglobin may cause staining of the dental surfaces, which could limit its clinical acceptability. Future research should focus on thoroughly evaluating the stability of Hb formulations, as well as investigating alternative sources or recombinant production methods to ensure both safety and reproducibility.

Regarding the surface hardness change, the groups treated with 1.0 mg/mL Hb, 1.0 mg/mL Hb + 0.5% STMP, 1.0mg/mL Hb + 1.0% STMP,1.0 mg/mL Hb + 3.0% STMP, and Elmex Erosion Protection® be effective for enamel surfaces; however, their protection mechanisms differ. The high binding strength of Hb to hydroxyapatite ^7^ explains its protective effect. Additionally, proteomic studies have confirmed the efficacy of Hb in protecting enamel ^(^ ^7^ ^,^ ^8^. The effect of STMP may be attributed to an increase in the retention of Ca^2+^ and F^-^ on STMP molecules adsorbed onto the enamel surface, rather than the deposition of CaF^2^ globules. In contrast, the protective mechanism of the commercial solution is associated with the combination of fluoride and tin, which react with hydroxyapatite, thereby reducing enamel solubility and protecting against demineralization. However, its long-term use can lead to discoloration of the enamel surface and an astringent sensation for the individual. This, coupled with the necessity for combining multiple components (SnCl^2^/NaF/AmF), renders it less advantageous compared to Hb associated with STMP.

While only the combination of Hb and STMP was tested in this study, it is important to consider the possible individual roles of each component in the observed protective effect. Hb, a high-molecular-weight protein with strong affinity for hydroxyapatite ^7^ ^,^ ^9^ ^,^ ^10^, likely enhances the acquired enamel pellicle, increasing this barrier against acid challenges ^7^. Notably, a previous study from our research group demonstrated that STMP alone did not provide significant protection against enamel erosion, underscoring the importance of its association with Hb for enhanced efficacy ^23^. When combined, Hb may reinforce the organic matrix of the pellicle. At the same time, STMP strengthens the inorganic mineral reservoir, which could explain the synergistic protective effect observed in the percentage surface hardness results.

Although the association of Hb with 0.5% STMP and 1.0% STMP yielded better results in terms of the percentage change in surface hardness, it was observed that isolated Hb provided the best protection in terms of %SRI, while the groups associated with STMP showed no statistically significant difference. This discrepancy may be attributed to the fact that, when treatments are effective, the change in reflection is minimal, as the eroded surface tends to reflect light from the equipment similarly. On the other hand, hardness is more susceptible to changes than surface reflection, as it is a more sensitive analysis.

The erosive protocol employed in this study was used for the initial screening of potential anti-erosive compounds; however, it has certain limitations. While it serves as a valuable tool for initial inquiries ^17^, it fails to replicate clinical scenarios completely. The primary constraint arises from the utilization of a static model, wherein the specimens are exposed to the same saliva for 2 hours. In real clinical settings, saliva is consistently produced and cleared away, potentially leading to variations in adsorption patterns and protective efficacy. Thus, in controlled laboratory settings, its use has inherent limitations that must be critically evaluated, especially considering the dynamic oral environment. It is worth mentioning that this study is the first to evaluate the potential protective effects of Hb in association with an inorganic component. For this reason, we choose treatments with a duration of 2 hours. Although the duration of the total erosive challenge (30 seconds) may seem limited, the encouraging outcomes pave the way for further investigations involving prolonged erosive challenges and treatments for shorter durations.

The following steps involving Hb and STMP will entail *in situ* and *in vivo* protocols, incorporating prolonged erosive challenges along with abrasive ones to evaluate the clinical effects. Ultimately, our results indicate that Hb combined with STMP demonstrated a preventive effect against erosive tooth wear.

The association between the organic component (Hb) and inorganic (STMP) has a synergistic effect, protecting against initial dental erosion in this protocol. These results gain strength from the combination of organic and inorganic components in new dental products related to controlling dental erosion through acquired pellicle engineering.
